# Chromothripsis: Basis of a Concurrent Unusual Association between Myelodysplastic Syndrome and Primary Ciliary Dyskinesia

**DOI:** 10.1155/2014/149878

**Published:** 2014-09-01

**Authors:** Abhinav Agrawal, Anar Modi, Sayee Sundar Alagusundaramoorthy, Wael Ghali

**Affiliations:** Department of Internal Medicine, Monmouth Medical Center, Long Branch, NJ 07740, USA

## Abstract

A 20 year old male was initially diagnosed suffering from Primary ciliary dyskinesia with symptoms of bronchiectasis, severe frontal, maxillary and ethmoid sinus disease. At the age of 20, the patient was also diagnosed with Myelodysplastic syndrome requiring Bone marrow transplant due to the advanced stage at time of presentation. Primary ciliary dyskinesia and Myelodsyplastic syndrome are both rare clinical conditions found in the general population, especially in young adults. This rare combination of disorders has never been reported in literature to the best of the author's knowledge. The presence of an advanced cancer and a genetic abnormality due to two deletions occurring in two arms of the same chromosome can be explained on the base of chromothripsis. A number of evidences have been published in the literature, about multiple deletions in chromosome 5 and advanced stages of MDS being associated with chromothripsis however this is the first case report on two deletions in chromosome 7 giving rise to two different clinical entities requiring multiple modes of management.

## 1. Introduction

The myelodysplastic syndromes (MDS) comprise a heterogeneous group of malignant hematopoietic stem cell disorders characterized by dysplastic and ineffective blood cell production and a variable risk of transformation to acute leukemia. These disorders may occur de novo or arise years after exposure to potentially mutagenic therapy (e.g., radiation exposure, chemotherapy). MDS occurs in 3-4 individuals per 100,000 in the US population. Prevalence increases with age. For instance, in individuals aged 60 and above, prevalence is 7–35 per 100,000 [1]. Other series have reported higher rates [2] but those studies were based on Medicare reporting and not on histopathological diagnoses.

Primary ciliary dyskinesia is an autosomal recessive genetic disorder characterized by the impairment of ciliary motility in the ciliated epithelial lining cells resulting in defective mucociliary clearance in the upper and lower respiratory tract, paranasal sinuses, eustachian tube, middle ear, and fallopian tubes. Common manifestations include bronchiectasis, rhinosinusitis, situs inversus, and fertility problems [3, 4]. The total number of individuals with PCD in the United States is estimated at 12,000 to 17,000 [5]. Commonly quoted figures for prevalence are between 1 : 4000 and 1 : 40 000, with the true prevalence probably about 1 : 10 000 [6, 7].

The concurrent occurrence of both myelodysplastic syndrome and primary ciliary dyskinesia has never been reported in the same patient in literature to the best of the authors' knowledge.

## 2. Case Presentation

**Figure 1:**
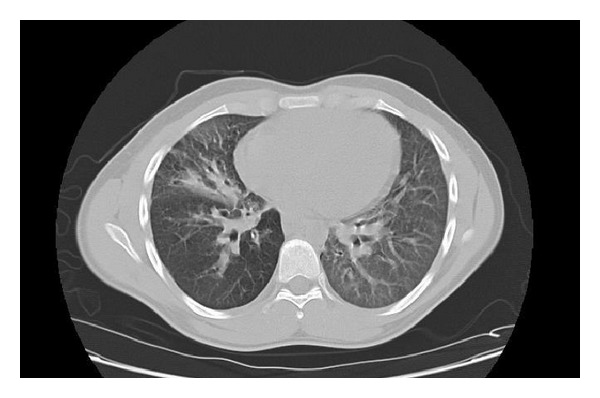
CT scan of the chest showing central bronchiectasis.

An 20-year-old male presented to the hospital with chief complaints of fever, chills, and shortness of breath. The chest radiograph at admission showed right middle lobe pneumonia with heavy growth of* Streptococcus pneumoniae* from his sputum without any bacteremia in his blood. On further evaluation, CT scan of the chest revealed right middle lobe and lingular infiltrate with evidence of central bronchiectasis (Figure 1) and CT scan of the sinuses showed severe congestion and inflammation of the frontal, maxillary, and ethmoid sinuses. The pulmonary function test showed predominantly obstruction of the small airways with no improvement after albuterol administration. A marked decrease in the diffusion capacity of the lung with a restrictive pattern was also noted. Further tests were ordered to explore the possibility of a multisystem disease and semen analysis showed a marked absence in motility of the sperm, thereby explaining PCD to be the probable cause of all his symptoms.

Biopsy of the nasal mucosa was obtained to prove the preliminary diagnosis and to obtain tissue for genetic testing. On electron microscopy of the biopsy specimen most of the cilia present in the epithelial cells of the ciliated epithelium lacked the inner dynein arms and some cilia lacked both dynein arms, thereby confirming the diagnosis of PCD.

During the subsequent visit for followup, the patient complained of worsening of his shortness of breath without any response to medical and respiratory therapy. At this time the patient was also found to have pancytopenia with platelet count of 57,000 cells/cu*·*mm, WBC count of 4,700 cells/cu*·*mm, and hemoglobin was 7.2 g/dL. Vitamin B12 and folate levels were normal and no source of infection could be identified after repeated tests and serial scans. Serology for Epstein-Barr virus, HHV1, HHV2, and mumps was negative. The workup for anemia only revealed a low reticulocyte count indicating bone marrow failure being the probable cause of pancytopenia. The patient underwent a bone marrow biopsy to identify the cause of marrow failure in the absence of any infection which revealed high-grade dysplasia and atypical localization of immature precursor cells in the bone marrow clinching the diagnosis of myelodysplastic syndrome (MDS). The bone marrow aspirate was sent for flow cytometry analysis and the results showed deletion of the D7S522 region in the long arm of chromosome 7 (also deletion of D20S108 of chromosome 20 at q12) characteristic of myelodysplastic syndrome or an evolving acute myeloid leukemia. Also observed in flow cytometry was a deletion in the short arm of chromosome 7 at sites 7p14-21, responsible for protein DNAH11, one of the proteins involved in the assembly of the dynein arm. FISH analysis was also done on this patient and was observed to be in concordance with flow cytometry results. Further cytogenetic testing did not reveal the presence of any further mutations. This concurrent presence of two deletions in the same chromosome helped to understand the probable etiology in development of these two distinct rare conditions simultaneously and their presentation in such a short rapid time interval. IPSS-R score was 4, classified as intermediate with a median survival time of 3 years and time for 25% of patients to progress to AML: 3.2. The severity of the disease processes warranted referral to a bone marrow transplant center where he was promptly pretreated with Fludarabine and Busulfan and underwent homologous stem cell transplant from his HLA compatible brother.

Regular followup at the tertiary care center showed good response of the transplanted bone marrow without any evidence of graft versus host disease (GVHD). He is also regularly followed up by a pulmonologist for management of his PCD.

## 3. Discussion

Primary ciliary dyskinesia and myelodysplastic syndrome are rare clinical conditions by themselves and have never been reported to occur together in the same patient in the literature. Of added significance is the fact that the patient was found to have MDS at the age of 20. The incidence of MDS in this age group, 20–24 years, is 0.1% of the the total incidence of MDS across all age groups [8].

**Figure 2:**
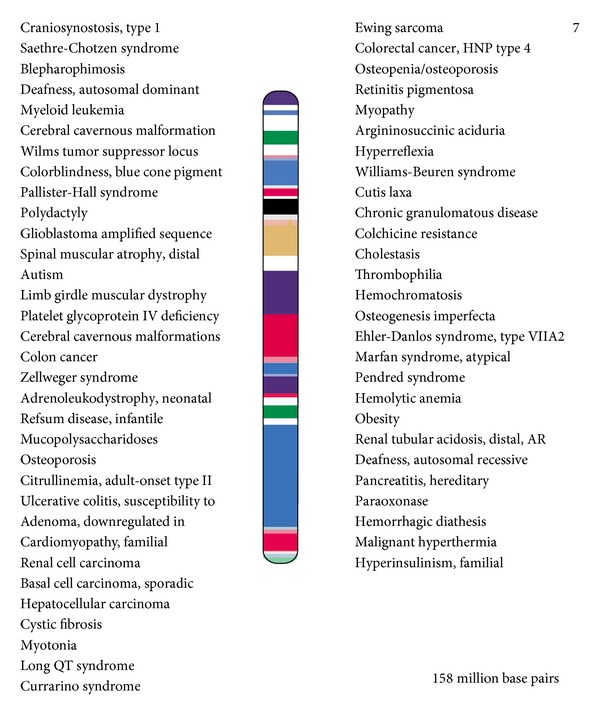
Cytogenetic disorders associated with chromosome 7.

Flow cytometry studies documented multiple deletions in chromosome 7 in this patient. MDS is seen to be associated with 7q31 deletion and PCD with 7p21. The occurrence of more than one deletion in the same chromosome has been well documented in the literature in chromosome 5 and observed clinically with Cri Du Chat syndrome associated with multiple other abnormalities. The most common cause attributed to such deletion is paternal pericentric inversion of chromosome 5. Such pericentric inversions of chromosome 5 tend to present at birth and are very rarely progressed, undiagnosed to a later age [9–12]. Zemanova et al. [13] reported complex chromosomal aberrations with nonrandom gene changes related to the advanced stages of MDS in bone marrow cells of 157 patients with newly diagnosed MDS. Chromothripsis and further chromosome shattering was present in 47% of the patients in this study. These patients also had a poor overall survival. The presence of advanced myelodysplastic syndrome at this age and the concurrent presence of primary ciliary dyskinesia can be attributed to this instability, presence of cryptic unbalanced rearrangements in chromosome 7. A single catastrophic event due to the inherent instability of the chromosome leading to the advanced stage of MDS and concurrent primary ciliary dyskinesia resulted from several complex genomic rearrangements and the changes arose all at once, the alteration of the genome occurring in a single event rather than in incremental steps [14, 15]. This presence of chromosomal instability and chromothripsis identifies a rare and aggressive entity of MDS with a poor survival and concurrent occurrence of other genetic abnormalities of chromosome 7. Both primary ciliary dyskinesia [16] and myelodysplastic syndrome have known to have been associated with chromosome 7. The various other cytogenetic disorders associated with chromosome 7 have been illustrated in Figure 2. Our patient did not have an occupational exposure to pesticides or organic solvents, which has also been mentioned to serve as a cause of 7p14 mutation.

Patients with MDS usually present with symptoms of fatigue, weakness, chest pain, or shortness of breath. Less commonly a person is diagnosed as a result of an infection, easy bruising, or bleeding. Initial diagnostic workup for MDS includes obtaining the complete blood count. Bone marrow aspiration and biopsy can be performed to achieve a definitive diagnosis [17]. Classification of MDS is done using the original and the revised International Prognostic Scoring System (IPSS & IPSS-R) and treatment recommendations are based on the risk group according to the scoring system [18, 19]. The only cure of MDS is blood or bone marrow transplantation. The following is the summary of options and recommendations for specific subsets of patients [20]. Patients with low risk MDS are treated with low intensity therapy including blood transfusions, hematopoietic growth factors [21], and low intensity chemotherapy using Azacitidine, Decitabine [22], and Lenalidomide [23]. Patients with high risk MDS are treated with either high risk chemotherapy similar to treatment of acute myeloid leukemia [24] or bone marrow transplantation [25]. Our patient was classified as intermediate risk according to the IPSS-R classification and underwent bone marrow transplantation because of the age and survival benefit.

Primary ciliary dyskinesia presents with recurrent infections of the upper and lower respiratory tract. Patients present with bronchiectasis manifesting as auscultatory crackles and may have wheezing. Common findings on chest radiograph and CT scan are mild to moderate degree of hyperinflation, peribronchial thickening, atelectasis, and bronchiectasis [26]. Spirometry often reveals mild to moderate airway obstruction with variable responsiveness to bronchodilators [27]. Rhino sinusitis is a cardinal feature of PCD commonly involving ethmoidal and maxillary sinuses. Assessment and followup of the sinuses should be done by sinus CT scan. Situs inversus is present in approximately 50% of patients with PCD. Most men with PCD have live but immotile spermatozoa and hence infertile. Measuring nasal nitric oxide (nNO) using chemoluminescence is a useful screening test for PCD but is limited to highly specialized centers [28]. High-speed video-microscopy (HSVM) and electron microscopy are traditional methods to examine ciliary movement and ultrastructure but requires experienced video microscopist and is very subjective [29]. Treatment of the disease must be highly individualized based on the needs and specific clinical course of the disease observed in each patient. Daily chest physiotherapy is very essential to compensate for absent mucociliary clearance. Acute exacerbations are commonly treated with antibiotics [30]. Bilateral lung transplantation is the therapy of choice in patients with end-stage respiratory insufficiency; often heart-lung transplantation or a modified surgical procedure is required since patients also present often with situs inversus [31]. Medical therapy for rhinosinusitis in patients with PCD includes nasal saline lavage, intranasal glucocorticoids, and antibiotic therapy for exacerbations. Close followup is essential and spirometry with pulmonary function tests should be obtained at every visit.

DNAH11 gene encodes a ciliary outer dynein arm protein and is a member of the dynein heavy chain family. It is a microtubule-dependent motor ATPase and has been reported to be involved in the movement of respiratory cilia. Bartoloni et al. [32] determined that the DNAH11 gene is composed of 82 exons extending over 353 kb of genomic sequence and maps to chromosome 7p21. Schwabe et al. [33] stated that full-length DHAH11 contains 4,523 amino acids. DHAH11 has an N-terminal domain, followed by 4 AAA domains, a helix-1-MTB-helix-2 domain, 2 additional AAA domains, and a C-terminal domain containing a conserved GVALL motif. Each of the first 4 AAA domains contains a P-loop motif predicted to mediate ATP hydrolysis. The helix-1-MTB-helix-2 domain is predicted to interact with a microtubule. In a boy with primary ciliary dyskinesia, Bartoloni et al. [32] identified a homozygous mutation in the DNAH11 gene. In addition to respiratory problems, the patient had dextrocardia with a structurally normal heart and visceral situs inversus, consistent with a diagnosis of Kartagener syndrome. In affected members of a German family with PCD, Schwabe et al. [33] identified compound heterozygosity for 2 mutations in the DNAH11 gene. One of the patients had Kartagener syndrome. Knowles et al. [34] sequenced DNAH11 in patients with a PCD clinical phenotype without a known genetic etiology and found that 69% had nonsense, insertion/deletion or loss of function splice site mutation and 22% had biallelic mutations in DNAH11.

The presence of complex chromosomal rearrangements has been well documented in the literature and is known to occur in patients with advanced stages of MDS and is usually associated with poor prognosis. This is the first documented evidence of the presence of a genetic abnormality with an advanced cancer as a result of the inherent instability and complex chromosomal rearrangements that happen in tumor cells. The presence of another concurrent genetic abnormality due to this rearrangement has not been published in the literature so far to the best of the authors' knowledge. This interesting combination of two distinct gene abnormalities of the same chromosome that occurred together in this patient is postulated to be the result of a single catastrophic event, throwing into light the mechanism of chromothripsis. The patient had no symptoms prior to this presentation and was apparently normal when both these diseases presented together due to the genetic rearrangements resulting from a single catastrophe. This complex chromosomal rearrangement represents the upper limit of survival for this cell and is essential for the cell to survive.

Hence, it becomes absolutely necessary that all the genetic abnormalities in any patient with chromosomal rearrangements are thoroughly investigated as the overall prognosis and survival of the patient depends on the degree of chromosomal rearrangements. Further research and more evidence of genetic abnormalities associated with multiplication of mutated tumor cells are needed to prove that chromothripsis can give rise to genetic abnormalities.
